# The Role of Thyrotrophin Receptor Antibody Assays in Graves' Disease

**DOI:** 10.1155/2012/525936

**Published:** 2012-04-19

**Authors:** C. Kamath, M. A. Adlan, L. D. Premawardhana

**Affiliations:** ^1^Department of Medicine, Caerphilly Miners' Hospital, St. Martin's Road, Caerphilly CF83 2WW, UK; ^2^Centre for Endocrine and Diabetes Sciences, University Hospital of Wales, Heath Park, Cardiff CF14 4XN, UK

## Abstract

Thyrotrophin receptor antibodies (TRAb) exist as stimulating or blocking antibodies in the serum (neutral TRAb have been identified recently). The clinical features of GD occur when stimulating TRAb predominate. But the relationship of TRAb to clinical phenotype and outcome is not clear when current assay methods are used. Therefore no consensus exists about its utility in diagnosing and predicting outcome in GD. The most commonly used TRAb assays, measure thyroid binding inhibiting immunoglobulins (TBII or “receptor assays”) and don't differentiate between stimulating and blocking antibodies. However, the more expensive, technically demanding and less freely available “biological assays” differentiate between them by their ability to stimulate cyclic AMP or failure to do so. Failure to differentiate between TRAb types and its heterogeneous molecular and functional properties has limited TBII use to GD diagnosis and differentiating from other forms of thyrotoxicosis. The current 2nd-3rd generation receptor assays are highly sensitive and specific when used for this purpose. TRAb assays should also be done in appropriate pregnant women. Current data do not support its use in outcome prediction as there is a significant variability of assay methodology, population characteristics and study design in published data, resulting in a lack of consensus.

## 1. Introduction

The immunopathogenesis of Graves' disease (GD) is a story that continues to evolve. GD is unique amongst autoimmune endocrine diseases as the underlying immune perturbation results in thyroid stimulation rather than its functional or structural inhibition. The contribution of genetic (MHC, CTLA-4, and PTPN22) and environmental influences (smoking, stress, drugs, micronutrients) to the aetiology of GD has been described extensively [1–6]. This complex genetic/environmental interaction results in the production of Thyrotrophin Receptor Antibodies (TRAb) which stimulate the TSH receptor (TSHR) and are the proximate cause of GD. Their precise role in the extrathyroidal manifestations of GD is currently being investigated [7].

The earliest description of a thyroid stimulator in GD was by Adams and Purves in 1956 [8]. The discovery of this “long-acting thyroid stimulator (LATS)” led to further attempts to characterize it [9]. The target antigen for LATS was the TSHR [10], and research showed these “thyroid stimulators” in GD were in fact autoantibodies to the TSHR; that is, TRAb. The complex nature of the interaction between TSHR and TRAb has been elegantly demonstrated using advanced techniques, and the molecular and crystalline structure of TRAb has been described in detail [11–14]. It would seem intuitive therefore that measurement of TRAb, the proximate cause of GD and so intimately involved in its pathogenesis, would assist in its diagnosis and management. However, neither contention is consistently borne out in clinical practice. The relationship between TRAb measured using currently available assays and GD is complex and needs to be understood by clinicians if they are to be correctly interpreted in clinical practice.

Current assays detect TRAb in 95-96% of subjects with GD although only some can demonstrate their functional characteristics [15]. However, there is no consensus about its role in diagnosing and managing GD, and its utility in predicting outcome. The inherent functional properties of TRAb, the variability in study design, and assay methodology have contributed to this uncertainty.

## 2. The Structure of TRAb and Their Interaction with TSHR in GD

TRAb are heterogeneous in both molecular structure and biological activity with a propensity to change during the course of the disease. They may stimulate the TSHR (thyroid stimulating antibodies-TSAb) or block its activity (thyroid blocking antibodies-TBAb) [16]. The clinical phenotype is thus determined by the balance between their opposing actions-thyrotoxicosis when TSAb predominate, and hypothyroidism when TBAb predominate. Neutral TRAb have also been isolated recently and their role in GD is yet to be defined [17]. TSAbs probably undergo affinity maturation and bind TSHR with high affinity, although details are not accurately known [18, 19]. A new classification has been proposed for TRAb based on their ability to stimulate or block both classical cyclic AMP (cAMP) and nonclassical non-cAMP signalling pathways. This classification is functionally more accurate and intellectually more attractive [16].

The TSHR is a G protein-coupled receptor and has a molecular structure consistent with this. The extracellular component consists of a Leucine-rich repeat domain (LRD) and a hinge region (HR), which links to the 7 domain transmembrane and intracellular components. The increasingly important role and the structure and function of the HR are currently being defined [20, 21]. There have been major recent studies of the synthesis, post translational modification, shedding of the *α*-subunit and the effect of the unbound *α*-subunit on the TSHR [22–25]. The *α*-subunit appears to be the primary autoantigen for TRAb formation [23, 26].

TRAb, in common with TSH, bind to the concave surface of the LRD. Recent crystallization studies using the TSHR stimulating human monoclonal antibody M-22 have shown the importance of several residues on this concave surface to the binding process [27] which seemed to be specific to this antibody [13]. These residues may not be specific for native TSH signalling. After binding to the TSHR, TRAb stimulate cAMP-dependent signal transduction (and also non-cAMP-dependent signalling pathways) resulting ultimately in increased thyroid hormone secretion [28]. The clinical features of GD are thus produced when TSAb predominate. Predominant TBAb have the opposite effect.

## 3. Measuring TRAb

### 3.1. Assay Methodology and Sensitivity

There are two currently available methods for measuring TRAb [29].

1. “Receptor assays” using I^125^ labelled TSH are freely available commercially for clinical use.
2. “Bioassays” using cultured cells, which measure cAMP production as an indicator of TSHR stimulation or inhibition, are still most often used in a research setting (Table 1).

#### 3.1.1. Receptor Assays

Receptor assays measure “thyroid-binding inhibiting immunoglobulins” (TBIIs); that is, antibodies that block binding of TSH to an in vitro TSHR preparation and do not therefore differentiate between TSAb and TBAb in serum samples. Some who do not advocate routine testing of TRAb in GD insist that this is of minor consequence as clinical and biochemical features will identify functional characteristics of the predominant TRAb in a patient with GD. The lack of correlation between TRAb in these assays and the clinical and biochemical severity of GD and its outcome may indeed be related to this inability to differentiate between the functional properties of TRAb. They therefore do not accurately predict GD phenotype in every patient. These assays also have wide intermethod variability. It has been estimated that the interassay coefficient of variation between various commercially available assays is 15.2–21.6% [30]. They are commercially freely available and are easy to perform (Table 1). 

While first-generation TBII assays using porcine cells and bovine labelled TSH had a sensitivity of only 50–80% [31], second-generation assays using recombinant human TSHR are said to be 90–99% sensitive and 95–100% specific [32–34]. Third-generation assays using human monoclonal TSHR stimulating antibodies are said to be even better [35] with improved sensitivities (97%) compared to second generation assays (94%) in one study [36].

There are still a minority of individuals who have GD who remain TRAb negative even when modern TBII assays are used. They usually have mild disease, smaller goitres, and minimal RAI uptake on scintigraphy [37]. In a recent study only 1.4% of an untreated group of thyrotoxic patients were in this group when a third-generation assay was used [38]. It is speculated that they have intrathyroidal TRAb production which does not spill over to the circulation, or that even third-generation TBII assays are too insensitive. Fully automated TBII assays are now available and should improve their use [39].

#### 3.1.2. Biological Assays

Biological assays in contrast measure the ability of TRAb to stimulate or inhibit TSHR activity. They measure the production of cAMP when sera-containing TRAb are exposed to TSHR on cell preparations such as FRTL-5 or CHO [40, 41]. Therefore, they are able to differentiate between TSAb and TBAb. However, their sensitivity at predicting GD recurrence is still surprisingly poor as some studies indicate [42]. This may relate to inherent properties of TRAb (e.g., antibodies with both blocking and stimulating activities, very similar receptor-binding characteristics and affinity for the TSHR) or to antibodies that interfere with these assays that make results difficult to interpret. More recent bioassays using a luciferase reporter gene on cell lines expressing the TSHR are technically less demanding and more rapidly done [43, 44].

Assays utilising modified TSHR, substituting some amino acid residues from the luteinizing hormone receptor (LHR), have produced encouraging results. These chimaeric TSHR-containing assay systems, for instance using the Mc4 TSHR where amino acid residues 262–368 of the human wild type receptor have been replaced by residues 262–334 of the rat LHR, seem to perform well under experimental conditions [45, 46].

Biological assays are currently limited to research in many centres. Although they provide information about the functional status of TRAb, their use has been restricted because of expense, and technical expertise and time required to perform them. Furthermore, the current utility of TBII assays in association with clinical and biochemical features to predict the functional status of TRAb in GD confers on them an advantage over biological assays. However, with advancing technology some of the above disadvantages should be overcome [26].

### 3.2. TRAb Assays and Specificity

Current TRAb assays lack specificity and may be positive in other thyroid disease. Recent studies have shown that a significant minority with painless thyroiditis (9.2%) and subacute thyroiditis (6.7%), hypothyroidism (9%) and multinodular goitre (17.2%) is TRAb positive using receptor assays [36, 52]. The inability of current assays to functionally define TRAb may account for this lack of specificity.

## 4. TRAb in the Diagnosis of Thyrotoxic States

### 4.1. Establishing a Diagnosis of GD and Differentiating from Other Causes of Thyrotoxicosis

Some argue that TRAb assays are not necessary to diagnose GD and for its differential diagnosis from other causes of thyrotoxicosis. If clinical symptoms and signs are nonspecific, they advocate the use of radioiodine (RAI) scintigraphy to differentiate GD from other thyrotoxic states [53]. In some centres about 20% remained of “indeterminate origin” even after RAI scintigraphy [54, 55], despite a retrospective cost effectiveness analysis comparing ultrasound to radioiodine scintigraphy in GD, which found a high sensitivity (97.4%) and specificity (98.8%) for RAI with equally good positive and negative predictive values [56]. Some argue that assays for other antibodies such as thyroid peroxidase antibodies (TPOAbs), present only in about 80% of GD but which are easier to perform and freely and more cheaply available, could be used instead of TRAb. TPOAb has a low sensitivity and specificity in this context and therefore is not very helpful in our opinion. Thus RAI uptake scans and TPOAb assays are inadequate for routine clinical use for the differential diagnosis of thyrotoxic states.

GD is difficult to diagnose in the minority of patients where goitre, overt clinical features, and GO are absent. The proponents of TRAb agree that the availability of sensitive and easy to perform, comparatively cheap assays should make TRAb an essential tool in the diagnostic work-up. Its high sensitivity ensures that virtually all subjects with GD are picked up. This is important from a practical point of view in centres where first line therapy for GD and other thyrotoxic states differs. Most clinicians treat GD initially with thionamides, before giving RAI therapy for a recurrence [57]. They also treat toxic nodular disease (almost all TRAb negative) with RAI as first line therapy (usually after making them euthyroid with thionamides) [58]. The use of TRAb would therefore help this decision-making process at an early stage. There is also an economic argument for using relatively cheap TRAb assays without using more expensive and cumbersome thyroid scintigraphy. In centres where TRAb assays have been established as routine and are cheaper to do, this differential in expense is even greater. The current use of TRAb in diagnosing GD seems to be governed by tradition, expense, and the availability of suitable assays.

## 5. Special Situations

### 5.1. Pregnancy

GD is responsible for nearly 85% of the 0.1–0.4% of pregnancies that are complicated by hyperthyroidism [59, 60]. Transplacental passage of TRAb causes foetal or neonatal thyrotoxicosis in 1–5% of pregnancies in women with current or past GD [61]. In the majority of pregnant women, TRAb levels begin to decline at around 20 weeks of gestation because of gestational immune modulation; the immune milieu is consistent with the Th2 paradigm during pregnancy and the important roles of hormones and regulatory T cells in this process are not within the scope of this paper [62]. The persistence of high levels of TRAb in the third trimester (measured between 22–26 weeks) increases risk to the foetus and indicates the need for close monitoring in association with obstetricians and neonatal specialists. Some would limit third trimester TRAb testing only to those mothers who had high titres in the first trimester [63]. Although investigators have attempted to correlate TRAb activity in the mother and neonate with foetal and neonatal GD, there has been no consensus. Some investigators found maternal TRAb of >40 U/L (using human recombinant receptor assays) predicted neonatal GD [64]. Japanese investigators also found that in mothers who had RAI for GD, TRAb levels at delivery were significantly higher in those who delivered infants with neonatal hyperthyroidism compared to those who did not [65].

The current indications for TRAb testing in pregnancy are as follows [66].

- Current GD that is, those on thionamide therapy.
- Previous radioiodine treatment or surgery for GD even if euthyroid—2–10% risk of foetal and neonatal hyperthyroidism.
- Previous history of delivering an infant with neonatal hyperthyroidism.

Subjects who have had previous GD who are in remission (i.e., on no drug therapy), do not need TRAb testing as their euthyroid state implies the absence of significant levels of TRAb and therefore no risk to the foetus.

### 5.2. Immune Reconstitution Syndromes

Modern lymphocyte depleting agents such as Alemtuzumab (CAMPATH), an anti-CD52 monoclonal antibody, cause thyroid dysfunction in a significant minority of patients, as many as 30% when used to treat multiple sclerosis. This immune reconstitution syndrome may also occur in highly active antiretroviral therapy (HAART) for HIV infection, and bone marrow transplantation from a GD patient [67]. These subjects develop GD and have detectable TRAb. The mechanisms in Alemtuzumab and HAART induced GD seem to be naive CD4 T-cell expansion, while a graft versus host disease may account for it in bone marrow recipients [67].

### 5.3. Orbitopathy

A significant proportion of subjects with GD have clinically evident Graves' orbitopathy (GO), estimated to be between 30–50% in various studies. Sight threatening disease occurs in about 5% [68]. The coexistence of symptoms and signs of GD in the majority of them helps establish an accurate diagnosis.

However, TRAb assays are mandatory in two circumstances: (a) to diagnose the minority where GO occurs as an isolated disorder without symptoms or signs of GD and (b) rarely when GO occurs in a hypothyroid patient.

## 6. What Happens to TRAb When GD Is Treated?

Both thionamide therapy and thyroid surgery reduce TRAb in GD. Thionamides, reduce TRAb primarily by their immunomodulatory effects [69, 70]. Surgery does so by removing the antigen, TSHR [71], and possibly by T and B lymphocytes apoptosis following high level antigen release during surgery [25]. The effects of RAI therapy on TRAb are different. An initial rise in TRAb after RAI is followed by a gradual fall [72]. This initial rise is probably a result of the release of TSHR antigen following tissue destruction by RAI. RAI-induced inhibition of T regulatory cells (TReg) may also contribute [73]. The modulation of TRAb levels after the three modalities of treatment described above would suggest that the persistence of TRAb at significant levels would predict further recurrences. However, the story is far from clear.

## 7. TRAb in Predicting Recurrences of GD

The inability of currently available TRAb assays to predict remission and recurrences of GD remains a great shortcoming in this area. A prediction tool such as TRAb could spare patients from long and sometimes complicated drug regimes with potentially serious side effects. The ability to predict the course of GD would also facilitate early definitive therapy with RAI or surgery. Clinical utility at predicting recurrences was inadequate when using clinical data (goitre volume, family history of GD, age, gender, smoking, etc.) and biochemical/immunological data (thyroid hormone levels, TRAb levels, rate of TRAb decline during treatment, etc.) either singly or in combination.

Early attempts at using TRAb to predict remission of GD followed a meta-analysis which suggested that the absence of TRAb after antithyroid drug therapy predicted remission [47]. But the practical value of this analysis was questionable and limited as nearly 25% of subjects were misclassified [29]. Although large scale, well-powered prospective studies addressing this question are lacking, a brief examination of the data from the last decade for the use of TRAb as a predictor of GD outcome is warranted (Table 2).

The predictive value for TRAb at the assay diagnostic cutoff value of 1.5 U/L, was low and not of high clinical utility in an early study published in 2002 [74]. Subsequent studies attempted to use TRAb thresholds that were higher, and measured at various points during the course of the disease to improve its predictive value. A cutoff above 10 IU/L at 6 months increased the positive predictive value (PPV) to 97% in one study. But its negative predictive value (NPV) was too low for clinical utility [48]. In a subsequent multicentre prospective study it was found that within 2 years of stopping antithyroid drugs (ATD) 49% of 96 patients relapsed. In this study TRAb at a level of 10 U/L measured at 4 weeks after stopping ATD had a PPV 0f 83% and NPV of 62% (specificity 92%). But TSH also measured at 4 weeks after stopping ATD had a PPV of 70% and a negative predictive (NPV) value of 62% for a relapse [49]. Another study made use of the fact that thyroid peroxidase antibodies (TPOAb) which are detectable in GD may be used to advantage in combination with TRAb to increase the predictive value of a relapse. 71.8% of 131 patients with GD relapsed during followup for between 10–77 months [49]. The PPV for relapse was 100% when a cutoff of >5000 U/mL was used for TPOAb and of >6 U/L for TRAb (Table 2). Cappelli and his colleagues studied 216 patients with GD prospectively for 120 months. They measured TRAb at diagnosis and every 6 months thereafter for the duration of the study. TRAb at >46.5 U/L at diagnosis had a PPV of 52% and NPV 0f 77% and at >30.7 U/L at 6 months had a PPV of 53.2% and NPV of 79% for a relapse [50]. A study comparing human monoclonal antibody M22-based TRAb assays and second generation TRAb assays by Massart and colleagues was not conclusive either [51]. They measured TRAb after 18 months of antithyroid drug treatment and found that the newer M22-based assays did not improve the predictive value of relapse. They also commented on high intermethod variability.

Defining a consensus is therefore difficult and relates to several pertinent issues. The above studies were variable both in relation to TRAb assay methodology and study design. Some studies were retrospective (with all their associated problems) and some prospective. In the retrospective studies attempts were made to find the most sensitive and specific cutoff values for TRAb and in one its use in combination with TPOAb was examined. They were also variable in the timing of TRAb assay, being measured at diagnosis or at different points in the course of their disease. Furthermore, population genetics and iodine status may also have influenced these studies as they were done in geographically disparate areas. Therefore, it is our view that till further good quality evidence is forthcoming, TRAb assays seem a rather blunt tool to predict remission or relapse of GD using current methodology. 

## 8. Conclusions and Indications for TRAb Testing

The clinical utility of TRAb as an important tool in the differential diagnosis of thyrotoxic states is established in our opinion. Although some experts doubt its value in subjects with typical features of GD, we believe that TRAb assays should be done in all patients to positively establish a diagnosis and to help in differentiating between the various causes of thyrotoxicosis. Most such experts base their argument for selective TRAb testing, on the basis of cost, availability of assays, and traditional practice. However, TRAb measurements using modern 2nd-3rd generation receptor assays are increasingly more freely available, quickly done and cheap (certainly in high volume laboratories). They offer a greater advantage over TPOAb and thyroid scintigraphy, in terms of higher sensitivity and specificity, logistical considerations and cost savings. Furthermore, newer automated 3rd-generation assays provide excellent sensitivity and specificity with high PPV and NPV in subjects with biochemical hyperthyroidism [75].  Table 3 illustrates the current indications for performing TRAb tests.

However, its utility in predicting GD remission/relapse is still unproven. An ideal prediction tool would be easy and cheap to measure, sensitive with high PPV and NPV, when measured early in the course of the disease. The lack of large, reproducible, well-designed, prospective studies is a shortcoming in this area of thyroidology. Furthermore, the variability of study design, TRAb assay methodology, and target study populations in currently published studies, added to the variability of intrinsic molecular and functional characteristics of the TRAb molecule, make this aspect of GD management frustrating and lacking in consensus.

## Figures and Tables

**Table 1:** A comparison of TBII and biological assays.

	TBII assays	Biological assays
Advantages	Freely available commercially	Differentiate between stimulating and blocking activities of TRAb
	Relatively cheap	
	Easy to perform	
	Sensitive 2nd-3rd generation assays available	

Disadvantages	Do not differentiate between stimulating and blocking activities of TRAb	Most are technically complex and time consuming
	Lack absolute correlation with clinical phenotype	Relatively expensive
	No correlation with severity of illness	
	Lack predictive value for GD outcome	

TBII are easy to perform, cheap and are highly sensitive. They remain the preferred assay method of choice in clinical practice. Bioassays have the ability to differentiate between stimulating and blocking TRAb, but the utility of this property in day-to-day clinical practice is unclear. Furthermore, they require greater technical expertise to perform and currently are more expensive.

**Table 2:** Recent clinical studies examining the utility of TRAb assays in predicting GD outcome.

Author (year, (ref))	Assay (*n*)	Study design	TRAb cutoff value	% relapse	PPV %
Zimmermann- Belsing et al. (2002, [47])	TBII (129)	TRAb assays at diagnosis (122) and at withdrawal of drugs (129): median followup 18 months	1.5 U/L	45	49
Quadbeck et al. (2005, [48])	TBII (96)	TRAb assays done 4 weeks after withdrawal of drugs: followup for 2 years	1.5 U/L	49	49
			10 U/L		83
Quadbeck et al. (2005, [48])	Bioassay (96)	As above	1.5 U/L		49
					TSAb-51
Schott et al. (2007, [49])	TBII (131)	TRAb and TPOAb assays done 4.3 months (mean) after GD diagnosis	>2 and <6 U/L	71.8	66.7–90
			>6 + >5000		100
			>6 + >500		93.7–96
Cappelli et al. [2007, [50]]	TBII (216)	TRAb assays done at diagnosis and 6 monthly for 120 months	>46.5 U/L at diagnosis or	67.1	52%
			>30.7 U/L at 6 months		53.2
Massart et al. (2009, [51])	TBII (128)	TRAb assays compared after 18 months of treatment: 3-year followup	0.94–3.2 IU/L	48	53–66%

Most recent studies are small and retrospective. They were variable in their study design (e.g., timing of TRAb measurement), assay methodology and TRAb cutoff values used for analysis, and population characteristics (i.e., geographically disparate). Although there was a high relapse rate (45–71.8%), TRAb assay by itself had a poor PPV and was a poor predictor of relapse even when different cutoff values were used.

**Table 3:** Current indications for TRAb testing.

Indications for TRAb testing
Establishing diagnosis of GD and differentiating from other thyrotoxic states
Thyrotoxicosis complicating the Immune reconstitution syndrome (CAMPATH and HAART)
Euthyroid or unilateral orbitopathy
Orbitopathy with hypothyroidism
Pregnancy in women:
(a) currently on ATD therapy
(b) who have had previous ablative therapy (RAI or surgery)
(c) with previous children who had neonatal thyrotoxicosis
In the first trimester and at 22–26 weeks gestation

The current indications for TRAb testing are detailed above. Its use is limited to diagnostic indications. There is no clinical utility of TRAb in predicting outcome at present.
